# Supplementary material for: Molecular structure and function of myelin protein P0 in membrane stacking
Source: Sci Rep. 2019 Jan 24;9:642. doi: 10.1038/s41598-018-37009-4 (PMC6345808; doi:10.1038/s41598-018-37009-4)
Supplement: Supplementary file 1 — Supplementary Information [file 41598_2018_37009_MOESM1_ESM.pdf]

Supplementary information

Molecular structure and function of myelin protein P0 in membrane stacking

Arne Raasakka[1,2], Salla Ruskamo[2], Julia Kowal[3,4], Huijong Han[2], Anne Baumann[1,5], Matti Myllykoski[2], Anna Fasano[6], Rocco Rossano[7], Paolo Riccio[7], Jochen Bürck[8], Anne S. Ulrich[8,9] Henning Stahlberg[3] & Petri Kursula[1,2,†]

[1]Department of Biomedicine, University of Bergen, Bergen, Norway
[2]Faculty of Biochemistry and Molecular Medicine & Biocenter Oulu, University of Oulu, Oulu, Finland
[3]Center for Cellular Imaging and NanoAnalytics (C-CINA), Biozentrum, University of Basel, Basel, Switzerland
[4]Institute of Molecular Biology and Biophysics, Department of Biology, ETH Zurich, Switzerland
[5]Division of Psychiatry, Haukeland University Hospital, Bergen, Norway
[6]Department of Biosciences, Biotechnologies and Biopharmaceutics, University of Bari, Bari, Italy
[7]Department of Sciences, University of Basilicata, Potenza, Italy
[8]Institute of Biological Interfaces (IBG-2), Karlsruhe Institute of Technology, Karlsruhe, Germany
[9]Institute of Organic Chemistry, Karlsruhe Institute of Technology, Karlsruhe, Germany

[†]Corresponding author. E-mail: petri.kursula@uib.no

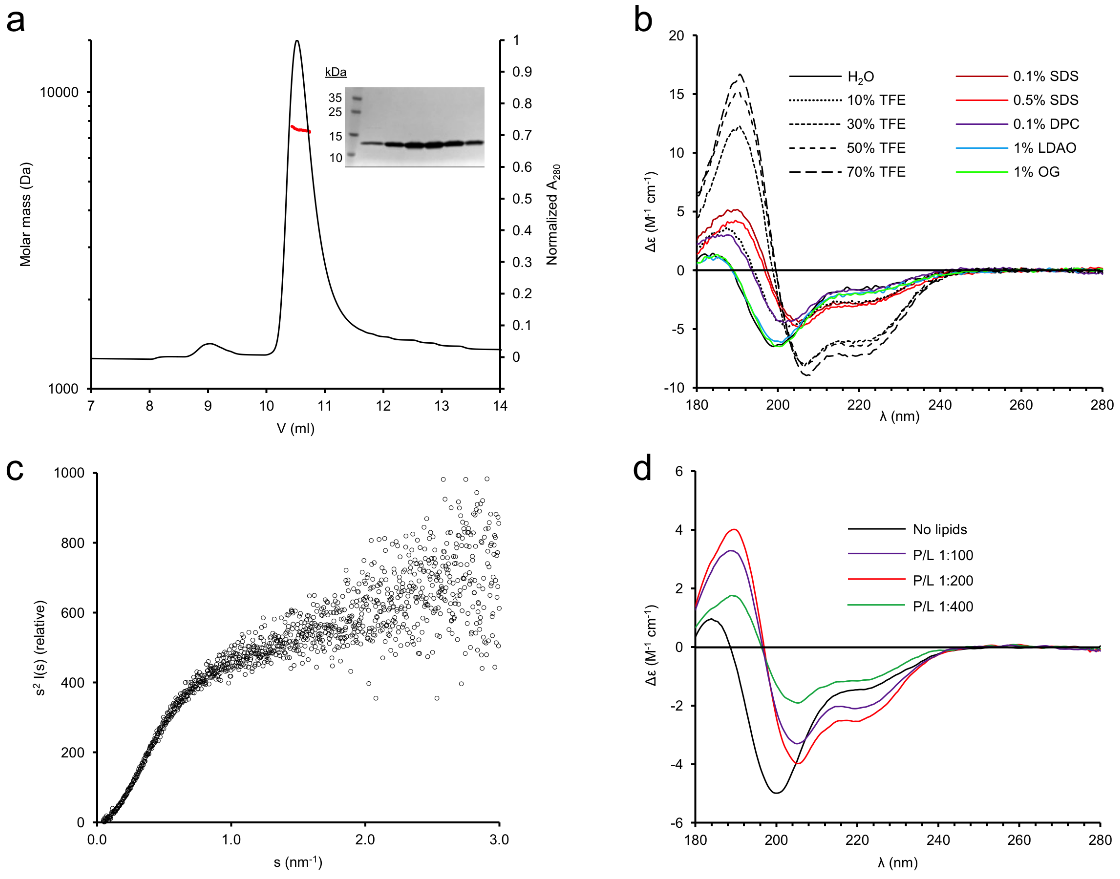

Supplementary Fig. S1. The purity, monodispersity, and folding of P0ct. (a) SEC-MALS profile of P0ct displays a mostly monodisperse preparation, with a single major peak corresponding to P0ct when eluting from a Superdex 75 10/300GL column. The calculated mass (7.5 kDa) matches that of a P0ct monomer. SDS-PAGE analysis of the purity of P0ct fractionated with a Superdex 75 16/60 HiLoad SEC column is shown as inset. (b) P0ct gains significant secondary structure content in TFE, DPC and SDS, but not in LDAO or OG, as shown using CD spectroscopy. (c) The Kratky plot displays the highly elongated nature of P0ct. (d) The SRCD spectra of P0ct in DMPC:DMPG (1:1) display different degrees of folding at different P/L ratios, 1:200 producing the strongest signal.

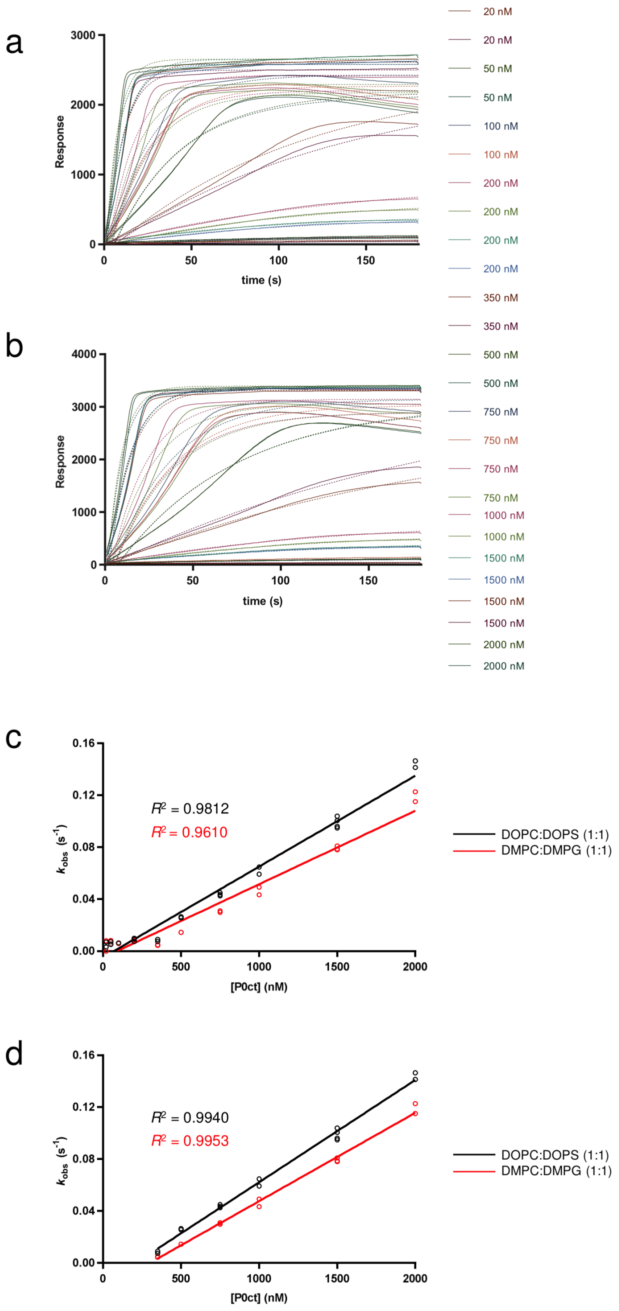

Supplementary Fig. S2. Kinetic analysis of SPR association data. The data (solid lines) of irreversible P0ct association with DOPC:DOPS (1:1) and DMPC:DMPG (1:1) (shown as panels a and b, respectively) vesicles fitted individually to an exponential one-phase binding model (dashed lines). (c) The derived k_{obs} values plotted against P0ct concentration, with the DOPC:DOPS (1:1) and DMPC:DMPG (1:1) data fitted to linear functions (see Supplementary Table 2 for the extracted k_{on} and k_{off} values). (d) Same data but all data points below the critical binding concentration have been omitted, resulting in a better linear fit.

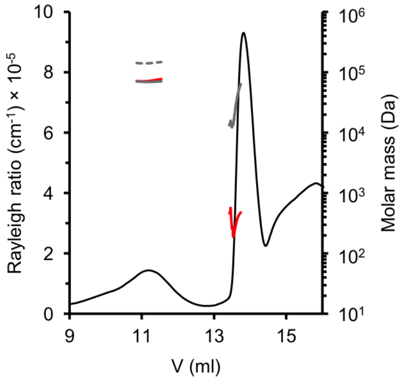

Supplementary Fig. S3. SEC-MALS of P0 in LDAO. Analysis of full-length P0 monodispersity and oligomeric state using SEC-MALS in LDAO. The Rayleigh ratio is shown (black) together with the total mass (gray dash), protein mass (red) and detergent mass for each peak (gray solid).

Supplementary Table S1. SAXS parameters.

**Data collection parameters**	
Instrument	P12, PETRAIII, DESY
Wavelength (nm)	0.124
Angular range (nm^{-1})	0.027 - 4.801
Exposure time (s)	0.045
Concentration range (mg ml^{-1})	1.1 - 4.2
Temperature (°C)	20
**Structural parameters**	
I_0 (relative) [from p(r)]	1882
R_g (nm) [from p(r)]	2.67
I_0 (relative) [from Guinier]	1892
R_g (nm) [from Guinier]	2.46
R_g (nm) [from EOM ensemble]	2.69
D_{max} (nm) [from GNOM]	11.06
D_{max} (nm) [from EOM ensemble]	8.03
**Molecular mass determination**	
Molecular mass M_r (kDa) [from I_0 using p(r)]	8.4
Molecular mass M_r (kDa) [from I_0 using Guinier]	8.4
Theoretical M_r from sequence (kDa)	7.99
**Software**	
Primary data reduction	PRIMUS
Data processing	PRIMUS
Ab initio analysis	GASBOR
Conformational ensemble analysis	EOM
Validation and averaging	PRIMUS
Three-dimensional graphics representation	PyMOL
**EOM model parameters**	
Conformer #1	
R_g (nm)	2.488
D_{max} (nm)	7.904
Mass fraction	0.182
Conformer #2	
R_g (nm)	2.867
D_{max} (nm)	8.264
Mass fraction	0.182
Conformer #3	
R_g (nm)	3.946
D_{max} (nm)	12.73
Mass fraction	0.182
Conformer #4	
R_g (nm)	2.068
D_{max} (nm)	6.14
Mass fraction	0.364
Conformer #5	
R_g (nm)	2.954
D_{max} (nm)	9.862
Mass fraction	0.090
Total mass fraction of main conformers	1.000

Supplementary Table S2. Kinetic parameters derived from P0ct association phase with vesicles.

Vesicle composition	Fitting set 1[a]			Fitting set 2[a]		
	k_{on} (nM^{-1} s^{-1})[b] × 10^5	k_{off} (s^{-1})[c] × 10^2	R^2	k_{on} (nM^{-1} s^{-1})[b] × 10^5	k_{off} (s^{-1})[c] × 10^2	R^2
DOPC:DOPS (1:1)	6.994 ± 0.1978	-0.4874 ± 0.1827	0.9812	7.873 ± 0.1629	-1.655 ± 0.1629	0.9940
DMPC:DMPG (1:1)	5.657 ± 0.2326	-0.5059 ± 0.2148	0.9610	6.804 ± 0.1252	-2.042 ± 0.1468	0.9953

[a] Fitting set 1 contains all data points from the linear fit, whereas all data points below 350 nM were omitted from Fitting set 2.

[b] Slope of the linear fit function to $k_{obs(on)}$ vs. [P0ct].

[c] Y-axis intercept of the linear fit function to $k_{obs(on)}$ vs. [P0ct]
